# Characterizing temperature-dependent optical properties of (MA_0.13_FA_0.87_) PbI_3_ single crystals using spectroscopic ellipsometry

**DOI:** 10.1038/s41598-019-54636-7

**Published:** 2019-12-03

**Authors:** Hsiao-Wen Chen, Desman Perdamaian Gulo, Yu-Chiang Chao, Hsiang-Lin Liu

**Affiliations:** 1 Department of Physics, National Taiwan Normal University, Taipei, 11677 Taiwan; 2 Department of Physics, Chung Yuan Christian University, Taoyuan, 32023 Taiwan

**Keywords:** Materials science, Condensed-matter physics, Electronic properties and materials

## Abstract

In this paper, we present spectroscopic ellipsometry measurements of (MA_0.13_FA_0.87_)PbI_3_ single crystals assessed at photon energies of 0.73–6.42 eV and at temperatures between 4.4 and 400 K. At room temperature, the refractive index was dispersed as a function of frequency, which is typical of a semiconductor. The absorption spectrum exhibited several electronic transitions. We estimated a room temperature direct band gap of 1.66 ± 0.02 eV and exciton binding energy of 40 meV. With decreasing temperature, the refractive index increased. The room-temperature thermo-optic coefficients were −1.7 × 10^−4^ and −2.5 × 10^−4^ K^−1^ at wavelength of 600 and 1200 nm. The exciton peak position and bandgap energy exhibited a redshift, which was attributed to a reverse ordering of the band structures. Additionally, an anomaly in exciton peak position and bandgap occurred at approximately 100–200 K due to the structural phase transition. This phenomenon was associated with the coexistence of MA/FA-disordered and MA/FA-ordered domains. Our results provide a foundation for the technological development of lead halide perovskites-based photonic devices at various temperatures.

## Introduction

Hybrid organic–inorganic lead halide perovskites have revolutionized the field of solution-processed photovoltaics^1–3^. The power conversion efficiencies of perovskite solar cells have been significantly improved, reaching up to 22.7%^4^. Large-scale commercialization of perovskite solar cells is possible pending the enhancement of the relevant material’s long-term stability in ambient conditions. Among the relevant materials, methylammonium lead triiodide (MAPbI_3_) and formamidinium lead triiodide (FAPbI_3_) have been most widely investigated. Studies have indicated that MAPbI_3_ exhibits thermal and moisture-related instabilities^5,6^. Due to these humidity-related and thermal instabilities of the constituents of MAPbI_3_, the unit cell of the perovskite breaks down and the constituents disintegrate to form other species, leading to the loss of the desirable photovoltaic properties. Regarding FAPbI_3_, studies have indicated that this material easily transforms into a yellow nonperovskite hexagonal structure^6,7^. A compositional engineering technique has been developed to enhance the stability of perovskite materials^8^. Specifically, MAPbI_3_ has been incorporated into FAPbI_3_, resulting in a stabilized perovskite phase that improves device efficiency and stability. (FAPbI_3_)_1 − x_(MAPbI_3_)_x_ material system shows a maximum power conversion efficiency value of 17.3% which was attributed to the increased short-circuit current density and fill factor^8^. The strong diffraction peak at 13.9^o^ remains even after annealing the (FAPbI_3_)_1 − x_(MAPbI_3_)_x_ film at 100 °C, which indicated the enhanced phase stability and crystallinity. *Ab initio* simulations also delivered a higher decomposition reaction energy for this perovskites^9^. Recent stability tests demonstrated a long lifetime more than 30 days of the device based on mixed organic cation based perovskite^10^.

For many of the practical applications of these materials, comprehensive studies of their optical properties are essential^11,12^. Furthermore, the temperature dependence of optical constants is a critical reference for determination of the effects of self-heating on device performance. Foley *et al*.^13^ examined the temperature-dependent photoluminescence, ultraviolet photoemission, and optical absorption spectra of MAPbI_3_ thin films. They discovered that the photoluminescence peak position and bandgap energy exhibited a redshift when temperature decreased from 358 to 301 K. This effect was attributed to the difference between the shifts of valence band maximum and conduction band minimum. Wright *et al*.^14^ and Dar *et al*.^15^ studied the temperature-dependent photoluminescence spectra of MAPbI_3_, MAPbBr_3_, FAPbBr_3_, and FAPbI_3_ thin films. The photoluminescence peak positions of these lead halide thin films exhibited a redshift as temperature decreased. Additionally, anomalous behavior of photoluminescence peak positions was observed at temperatures lower than 150 K due to structural phase transition. Jiang *et al*.^16^ studied the temperature-dependent spectroscopic ellipsometry spectra of MAPbI_3_ thin film. As temperature decreased, both the dielectric function and absorption spectra exhibited anomalies during the structural phase transition. Wu *et al*.^17^ reported the room-temperature photoluminescence and absorption spectra of MA(EA)PbI_3_ and FA(MA)PbI_3_ single crystals. They reported the values of bandgap energy for FA(MA)PbI_3_ and MA(EA)PbI_3_ as 1.46 and 1.49 eV.

Most optical measurements of lead halide perovskites have been limited to polycrystalline films. The temperature-dependent optical properties of lead halide perovskites single crystals have not been reported. In this study, we conducted a comprehensive optical study of (MA_0.13_FA_0.87_)PbI_3_ single crystals by using spectroscopic ellipsometry. The aim of the present study was to determine the temperature variation of the optical constants and the electronic structures of these materials.

## Experiment

**Figure 1:**
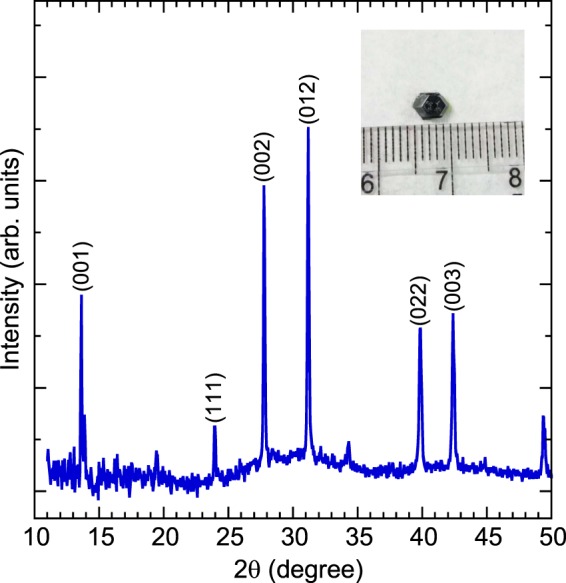
X-ray powder diffraction pattern of (MA_0.13_FA_0.87_)PbI_3_ at room temperature. The inset presents the optical microscopic image of a (MA_0.13_FA_0.87_)PbI_3_ single crystal.

Single crystals of (MA_0.13_FA_0.87_)PbI_3_ were prepared using an inverse temperature crystallization method. One molar perovskite precursor solution was prepared in γ–butyrolactone containing 0.027 g of cesium methylammonium iodide, 0.1427 g of formamidinium iodide, and 0.461 g of lead(II) iodide. The precursor was maintained in a vial in an oil bath at 88–91 °C under an ambient condition. (MA_0.13_FA_0.87_)PbI_3_ single crystals with the (001) surface were approximately 5 mm in diameter, as depicted in the inset of Fig. 1. Crystals of the same batch were characterized using x-ray powder diffraction. X-ray diffractometry data were obtained using a Philips X’pert Pro MRD diffractometer (Cu Kα radiation, λ = 0.15418 nm). The photoluminescence spectrum was recorded using a spectrometer (tecSpec MMS) with a 405-nm laser as an excitation source.

Room temperature spectroscopic ellipsometric measurements were conducted under angles of incidence between 60° and 75° and over a spectral range of 0.73 to 6.42 eV using a Woollam M-2000U ellipsometer. For the temperature-dependent measurements, the sample was placed in an ultrahigh-vacuum continuous-flow helium cryostat, enabling measurements at temperatures of 4.4–400 K. Due to the 70° angle of the two cryostat windows, only a single angle of incidence is possible. The raw ellipsometry data Ψ and Δ are related to the complex Fresnel reflection coefficients for light polarized parallel (*R*_*p*_) and perpendicular (*R*_*s*_) to the plane of incidence

1 $$\tan \,{\rm{\Psi }}{e}^{i{\rm{\Delta }}}=\frac{{R}_{p}}{{R}_{s}}.$$

To determine the complex refractive index of (MA_0.13_FA_0.87_)PbI_3_ single crystal, the experimental data were processed using a three medium optical model consisting of single crystal/surface roughness/air ambient structure. Then the error function σ was minimized in the entire spectral range

2 $${\sigma }^{2}=\frac{1}{m}\sum _{i=1}^{m}[{({{\rm{\Delta }}}_{\exp }-{{\rm{\Delta }}}_{calc})}^{2}+{({{\rm{\Psi }}}_{\exp }-{{\rm{\Psi }}}_{calc})}^{2}],$$

where Δ_calc_, Ψ_calc_ and Δ_exp_, Ψ_exp_ are, respectively, the calculated and experimental ellipsometric data and m is the number of points in the spectrum. The Lorentz approximation was used to fit the spectral dependence of Ψ and Δ and calculate the complex refractive index. The origin source of this method description can be found in ref. ^18^ The spectroscopic ellipsometry analysis fitting results are shown in the Supplementary Information. The surface roughness is around 10.8 nm and the mean square error (MSE) is 0.86.

## Results and Discussion

Figure 1 illustrates the room temperature x-ray diffraction pattern of (MA_0.13_FA_0.87_)PbI_3_. All of the reflections could be indexed, and no impurity phases were apparent above the background level. Additionally, the spectra matched well with those previously observed^19–22^. A previously study on mixed-halide perovskites reported peaks at 11.63° and 12.85°, which were attributed to the α-phase of FAPbI_3_ and cubic PbI_2_^19–22^. However, no such peaks were observed in the x-ray diffraction pattern in this study, indicating the high purity of our single crystals. Rietveld refinement results of (MA_0.13_FA_0.87_)PbI_3_ revealed a cubic structure belonging to the Pm-3m space group with the lattice parameter of *a* = 6.439 Å.

**Figure 2:**
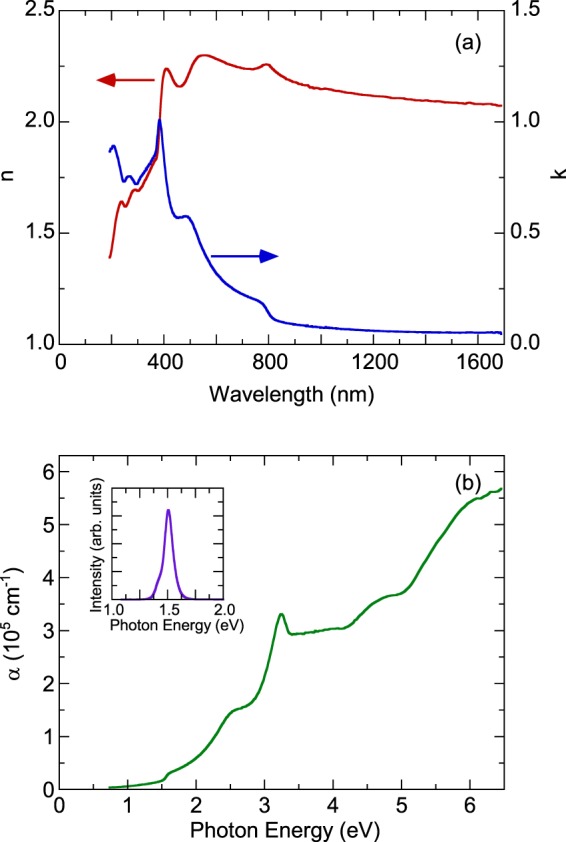
(**a**) Refractive index n and extinction coefficient k of (MA_0.13_FA_0.87_)PbI_3_ at room temperature. (**b**) Optical absorption coefficient of (MA_0.13_FA_0.87_)PbI_3_ at room temperature. The inset presents the room-temperature photoluminescence spectrum of (MA_0.13_FA_0.87_)PbI_3_.

Figure 2(a) presents the room temperature refractive index n and extinction coefficient k of (MA_0.13_FA_0.87_)PbI_3_, which were obtained through spectroscopic ellipsometry analysis. The room temperature experimental ellipsometric and best-fit calculated data of (MA_0.13_FA_0.87_)PbI_3_ were shown in the Supplementary Information. These data were almost identical by rotating the sample’s azimuthal orientation of 45° and 90° shown in the Supplementary Information, indicating the isotropic optical properties of the sample. Dispersion of the refractive index n as a function of frequency is typical of a semiconductor. Optical transitions were identified in the spectra according to resonance and antiresonance features that appeared at the same energy in k and n, respectively. Specifically, the extinction coefficient spectrum k of (MA_0.13_FA_0.87_)PbI_3_ was dominated by several optical transitions. We calculated the optical absorption coefficient *α* by *α* = *4πk/λ*, where *k* is the extinction coefficient and *λ* is the wavelength. Figure 2(b) displays the room temperature optical absorption spectrum of (MA_0.13_FA_0.87_)PbI_3_. In the inset of Fig. 2(b), we present the room-temperature photoluminescence spectrum, which exhibited a strong luminescence signal at approximately 1.50 eV. On the basis of previous reports^14,15^, Wright *et al*.^14^ presented the room temperature photoluminescence peaks of FAPbI_3_ and MAPbI_3_ at approximately 1.53 and 1.60 eV and Dar *et al*.^15^ found the room temperature photoluminescence peak of MAPbI_3_ at approximately 1.61 eV. The observed strong luminescence peak at approximately 1.50 eV for (MA_0.13_FA_0.87_)PbI_3_ is associated with the exciton transition.

**Figure 3:**
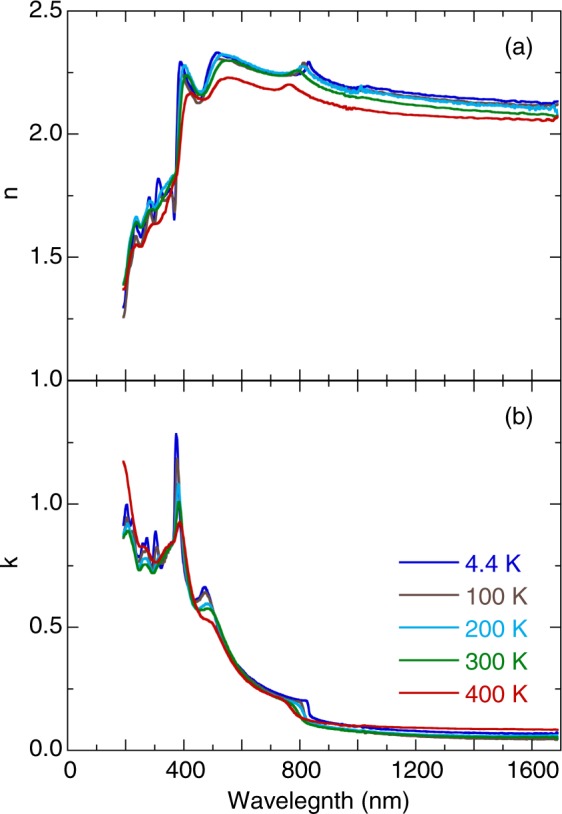
Temperature dependence of the (**a**) refractive index and (**b**) extinction coefficient spectra of (MA_0.13_FA_0.87_)PbI_3_.

**Figure 4:**
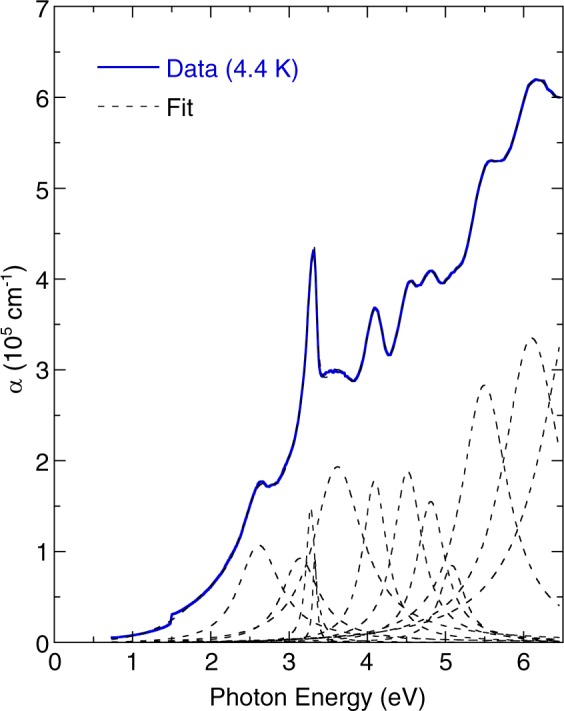
Optical absorption coefficient of (MA_0.13_FA_0.87_)PbI_3_ measured at 4.4 K. The dashed lines represent the best fit for the Lorentzian model.

Figure 3(a,b) illustrates the temperature dependence of the refractive index n and the extinction coefficient k of (MA_0.13_FA_0.87_)PbI_3_. As the temperature decreased, the refractive index increased in the visible and near-infrared-frequency region. The room-temperature thermo-optic coefficients of the refractive index (*dn/dT*) were approximately −1.7 × 10^−4^ and −2.5 × 10^−4^ K^−1^ at wavelength of 600 and 1200 nm. This behavior differs from that known for conventional semiconductors such as silicon, which shows a positive value for the thermo-optic coefficient^23^. According to Ghosh’s model^24,25^, *dn/dT* can be expressed as follows:

3 $$\frac{dn}{dT}=A(\,-\,3\alpha R-\frac{1}{{E}_{eg}}\frac{d{E}_{eg}}{dT}{R}^{2}),$$

where *A* = *(n*^2^ *− 1)/*2*n*, is the linear thermal expansion coefficient, *E*_eg_ is the excitonic bandgap, and *R* = *λ*^2^*/(λ*^*2*^ *−* ${\lambda }_{ig}^{2}$) is the normalized dispersive wavelength (*λ*_*ig*_: wavelength corresponding to the isentropic bandgap). In the case of halide perovskites, the first term is negative because the thermal expansion coefficient is positive, and the second term is negative due to positive *dE*_eg_*/dT*. As a results, (MA_0.13_FA_0.87_)PbI_3_ exhibits the negative value of the thermo-optic coefficient. A similar tendency for temperature dependence has been detected in the refractive index of TiO_2_ thin films^26^ and KTaO_3_ single crystals^27^. In the present study, the extinction coefficient spectra indicated that the absorption features became sharper and shifted to a higher energy. Figure 4 depicts the optical absorption spectrum of (MA_0.13_FA_0.87_)PbI_3_ at 4.4 K. This absorption spectrum was modeled reasonably well using the Lorentzian oscillators. The peak energy and assignment are summarized in Table 1. In accordance with previous reports^10–15^, the first absorption peak near 1.50 eV was assigned to the exciton transition. The absorption peaks at approximately 2.61, and 3.13 eV were associated with the charge-transfer excitations of the N *2p* orbital to the I *5p* orbital. The absorption peak at approximately 3.33 eV was attributed to the charge-transfer excitation of the I *5p* orbital to the Pb *5p* orbital. The absorption peaks near 3.62 and 4.09 eV were attributed to the charge-transfer excitations of the C *2p* orbital to the I *5p* orbital. Additionally, the absorption peaks at approximately 4.51, 4.81, 5.08, and 5.50 eV were associated with the charge-transfer excitations of I *5p* orbital to Pb *6p* orbital.

**Table 1:** Optical transitions observed at 4.4 K with their corresponding assignments.

Peak energy (eV)		Assignment
ω_1_	1.50	Exciton
ω_2_	2.61	N *2p* − I *5p*
ω_3_	3.13	
ω_4_	3.33	I *5p* − Pb *5p*
ω_5_	3.62	C *2p* − I *5p*
ω_6_	4.09	
ω_7_	4.51	I *5p* − Pb *6p*
ω_8_	4.81	
ω_9_	5.08	
ω_10_	5.50	

**Figure 5:**
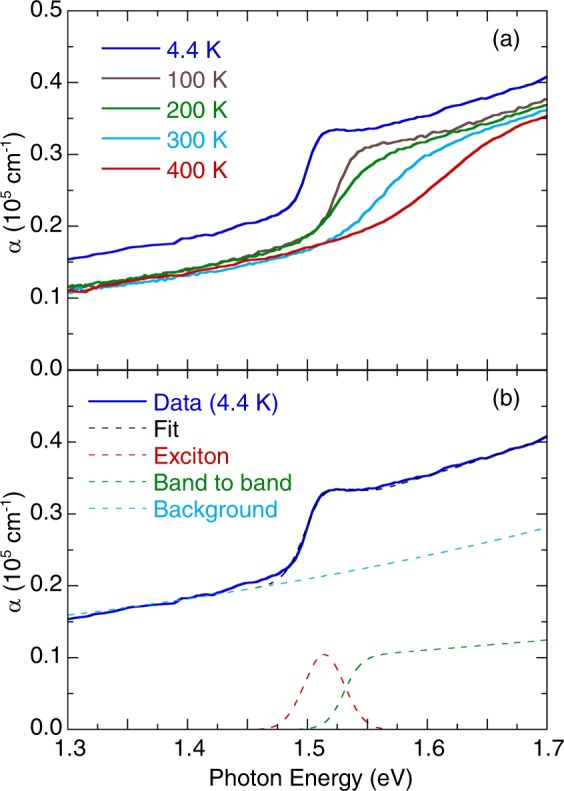
(**a**) Temperature dependence of (MA_0.13_FA_0.87_)PbI_3_ exciton absorption spectra. (**b**) The absorption edge of (MA_0.13_FA_0.87_)PbI_3_ measured at 4.4 K. The dashed lines are the best fit using the Elliott formula.

**Figure 6:**
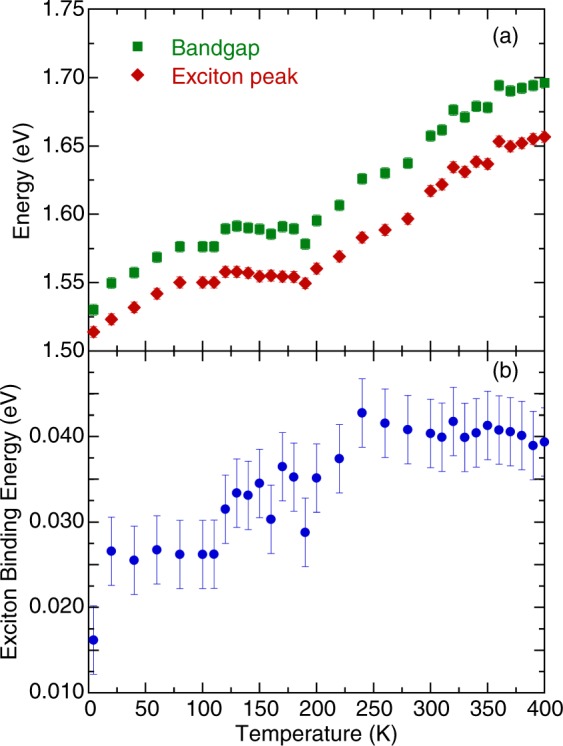
(**a**) Bandgap energy and peak position of exciton in (MA_0.13_FA_0.87_)PbI_3_ as a function of temperature. (**b**) Temperature dependence of exciton binding energy in (MA_0.13_FA_0.87_)PbI_3_.

In order to analyze the temperature-dependent exciton transition and bandgap energy, we have fitted the low-energy portion of the optical absorption spectra using the Elliott formula^28,29^

4 $$\begin{array}{ccc}\alpha (E) & = & [A\cdot \theta (E-{E}_{g})\cdot {D}_{CGV}(E)]\,[\frac{\pi x{e}^{\pi x}}{\sinh (\pi x)}]\\  &  & +\,A\cdot {R}_{x}\cdot \sum _{n=1}^{\infty }\frac{4\pi }{{n}^{3}}\cdot \delta (E-{E}_{g}+{R}_{x}/{n}^{2}),\end{array}$$

where *A* is a constant, *E* is the photon energy, *E*_*g*_ is the bandgap energy, *θ* is the step function, *D*_*CV*_ is the joint density of states described as *D*_*CV*_ ~ (*E* − *E*_*g*_) near the direct band edge, *n* is the principal quantum number of the exciton state, *δ* presents a delta function, and *x* = [*R*_*x*_/(*E* − *E*_*g*_)], with *R*_*x*_ being the exciton binding energy. Figure 5(a) presents the temperature-dependent exciton absorption spectra of (MA_0.13_FA_0.87_)PbI_3_. Figure 5(b) shows the absorption edge and excitonic contribution fitting results using the Elliott formula. The background was fitted with the standard Lorentzian profile. The values of *E*_*g*_ (≈1.53 and 1.66 eV) and *R*_*x*_ (≈16 and 40 meV) were determined at 4.4 and 300 K, respectively. Figure 6(a) denotes the temperature-dependent exciton peak position and bandgap energy of (MA_0.13_FA_0.87_)PbI_3_. The unusual redshift can be attributed to a reverse ordering of the electronic band structure^13^. Additionally, anomalous behavior in exciton peak position and bandgap energy was observed at 100–200 K due to the structural phase transition. This phenomenon was associated with the coexistence of MA/FA-disordered and MA/FA-ordered domains^15^.

The temperature-dependent exciton binding energy is shown in Fig. 6(b). At high temperature phase, the exciton binding energy of (MA_0.13_FA_0.87_)PbI_3_ is around 40 meV. It decreases to 16 meV at 4.4 K. Our room temperature results are close to the values predicted by the first-principles calculations (MAPbI_3_ ~ 45 meV and FAPbI_3_ ~ 35 meV)^30^. Notably, the exciton binding energy of (MA_0.13_FA_0.87_)PbI_3_ decreases when the temperature is lowered. This trend is similar with the previous absorption and photoluminescence measurements of MAPbI_3_ reported by S. Singh *et al*.^31^. The exciton binding energy of MAPbI_3_ was approximately 30 and 15 meV at high temperature tetragonal phase and low temperature orthorhombic phase, respectively. However, our results are different from the previous photoluminescence, photoabsorption, and magneto-transmission measurements in MAPbI_3_. Y. Yamada *et al*. reported the exciton binding energy of MAPbI_3_ to be approximately 6 and 30 meV at room temperature and 13 K, respectively^32^. A. Miyata *et al*. measured a value of the exciton binding energy of MAPbI_3_ to be a few meV at room temperature, but 16 meV in the low temperature orthorhombic phase^33^. Further theoretical work is needed to clarify the discrepancy of the experimental data.

**Figure 7:**
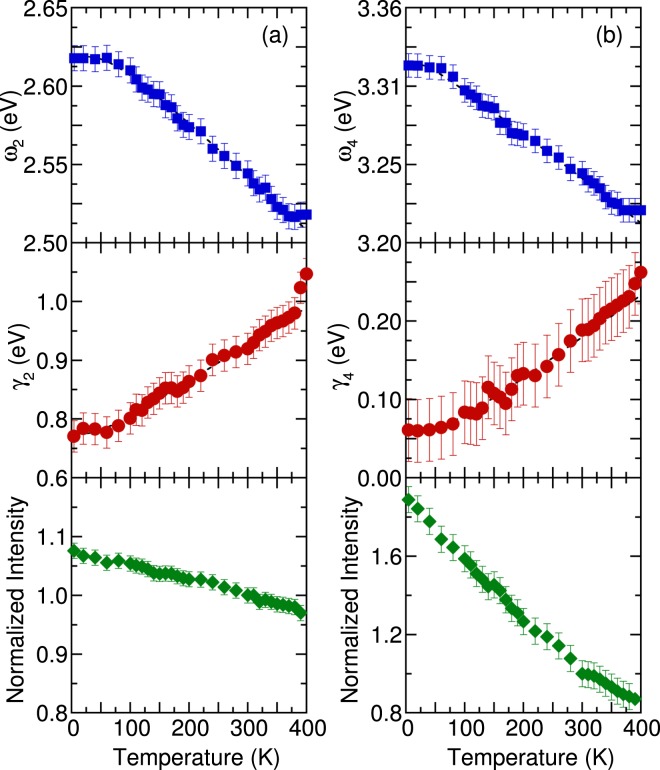
(**a**) Temperature dependence of peak energy, damping, and normalized intensity of (**a**) 2.61 and (**b**) 3.33 eV optical absorptions in (MA_0.13_FA_0.87_)PbI_3_. The dashed lines represent the fitting results of the Bose–Einstein model.

Figure 7 denotes the peak energy, damping, and normalized intensity of 2.61 and 3.33 eV optical transitions as a function of temperature. The temperature-dependent parameters of other optical transitions are shown in the Supplementary Information. The observed increase in the peak energy and decrease of damping with decreasing temperature can be described using the Bose–Einstein model^34^.

5 $$E=a-b[1+\frac{2}{{e}^{\frac{{\rm{\Theta }}}{T}}-1}],$$

and

6 $${\rm{\Gamma }}={{\rm{\Gamma }}}_{o}[1+\frac{2}{{e}^{\frac{{\rm{\Theta }}}{T}}-1}]+{{\rm{\Gamma }}}_{1}$$

where *a* and Γ_1_ are the transition energy and linewidth at 0 K; *b* and Γ_o_ represent the strength of the electron-phonon interactions; and Θ is the average phonon temperature. For the analysis of 2.61 eV electronic excitation, the values of *a* (≈2.62 eV), *b* (≈28 meV), Γ_1_ (≈0.72 eV), Γ_o_ (≈302 meV), and Θ (≈201 K) were determined. For the analysis of the 3.33 eV electronic excitation, the values of *a* (≈3.34 eV), b (≈12 meV), Γ_1_ (≈0.14 eV), Γ_o_ (≈112 meV), and Θ (≈240 K) were determined. The dashed lines in Fig. 7(a,b) represent theoretical predictions based on Eqs. (5, 6). These two optical transitions exhibited a slight deviation from the Bose-Einstein predictions to the temperature dependence of the peak position and linewidth between 100 and 200 K due to the structural phase transition.

## Summary

We investigated the temperature-dependent optical properties of (MA_0.13_FA_0.87_)PbI_3_ single crystals using spectroscopic ellipsometry. The absorption emerging in extinction coefficient spectrum indicated that (MA_0.13_FA_0.87_)PbI_3_ has a direct band gap of 1.66 ± 0.05 eV with a large exciton binding energy of 40 meV. With decreasing temperature, the refractive index increased. The room-temperature thermo-optic coefficients were −1.7 × 10^−4^ and −2.5 × 10^−4^ K^−1^ at wavelength of 600 and 1200 nm. The exciton transition and bandgap energy demonstrated sensitivity to the structural phase transition at 100–200 K. The data presented in this study offer insights into the design and fabrication of lead halide perovskites-based photonic devices for integrated optics and solar cell applications at various temperatures.

## Supplementary information

supplementary information
