# Supplementary material for: Characterizing temperature-dependent optical properties of (MA0.13FA0.87) PbI3 single crystals using spectroscopic ellipsometry
Source: Sci Rep. 2019 Dec 3;9:18253. doi: 10.1038/s41598-019-54636-7 (PMC6890668; doi:10.1038/s41598-019-54636-7)
Supplement: Supplementary file 1 — supplementary information [file 41598_2019_54636_MOESM1_ESM.pdf]

Supplementary information

Characterizing temperature-dependent optical properties of $(MA_{0.13}FA_{0.87})\,PbI_3$ single crystals using spectroscopic ellipsometry

Hsiao-Wen Chen[1], Desman Perdamaian Gulo[2], Yu-Chiang Chao[1], and Hsiang-Lin Liu[1,*]

[1]*Department of Physics, National Taiwan Normal University, Taipei 11677, Taiwan*

[2]*Department of Physics, Chung Yuan Christian University, Taoyuan 32023, Taiwan*

*Corresponding author: hliu@ntnu.edu.tw

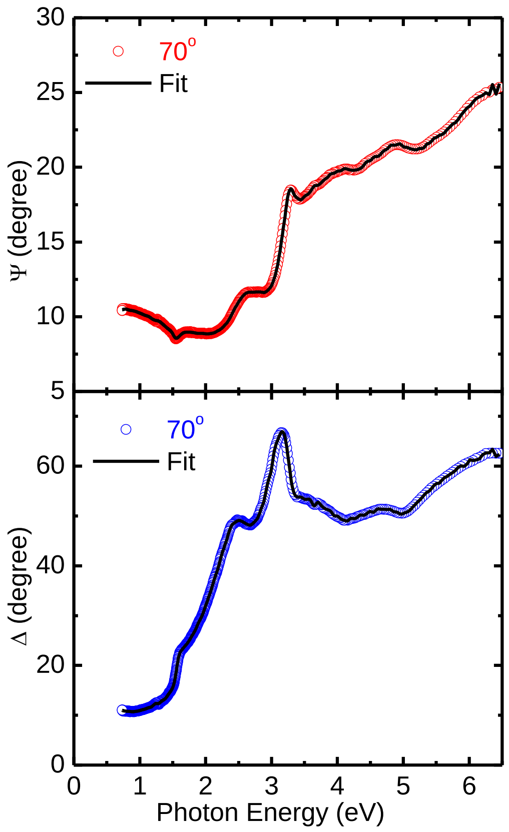

FIG. S1 Room-temperature experimental at 70° incidence angle and fitting model of ellipsometric parameters of psi (Ψ) and delta (Δ) of $(MA_{0.13}FA_{0.87})PbI_3$.

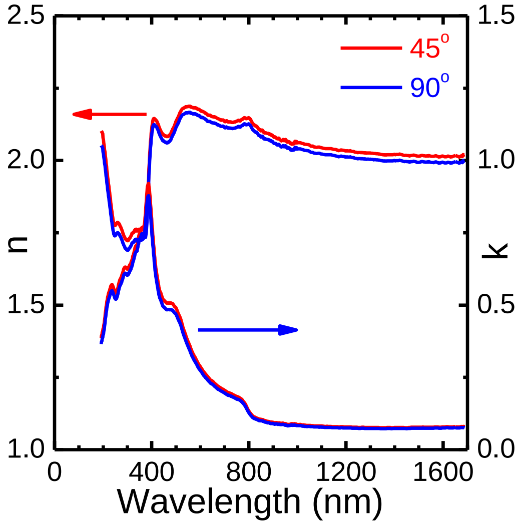

FIG. S2 Room-temperature refractive index n and extinction coefficient k of $(MA_{0.13}FA_{0.87})PbI_3$ by rotating the sample's azimuthal orientation by 45 degree and 90 degree.

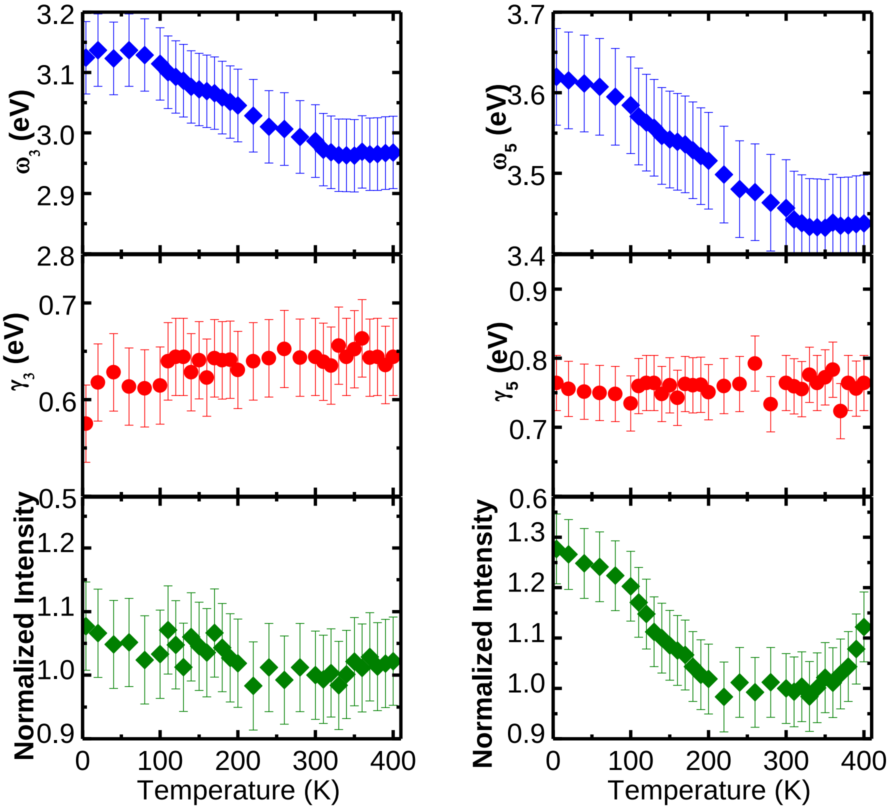

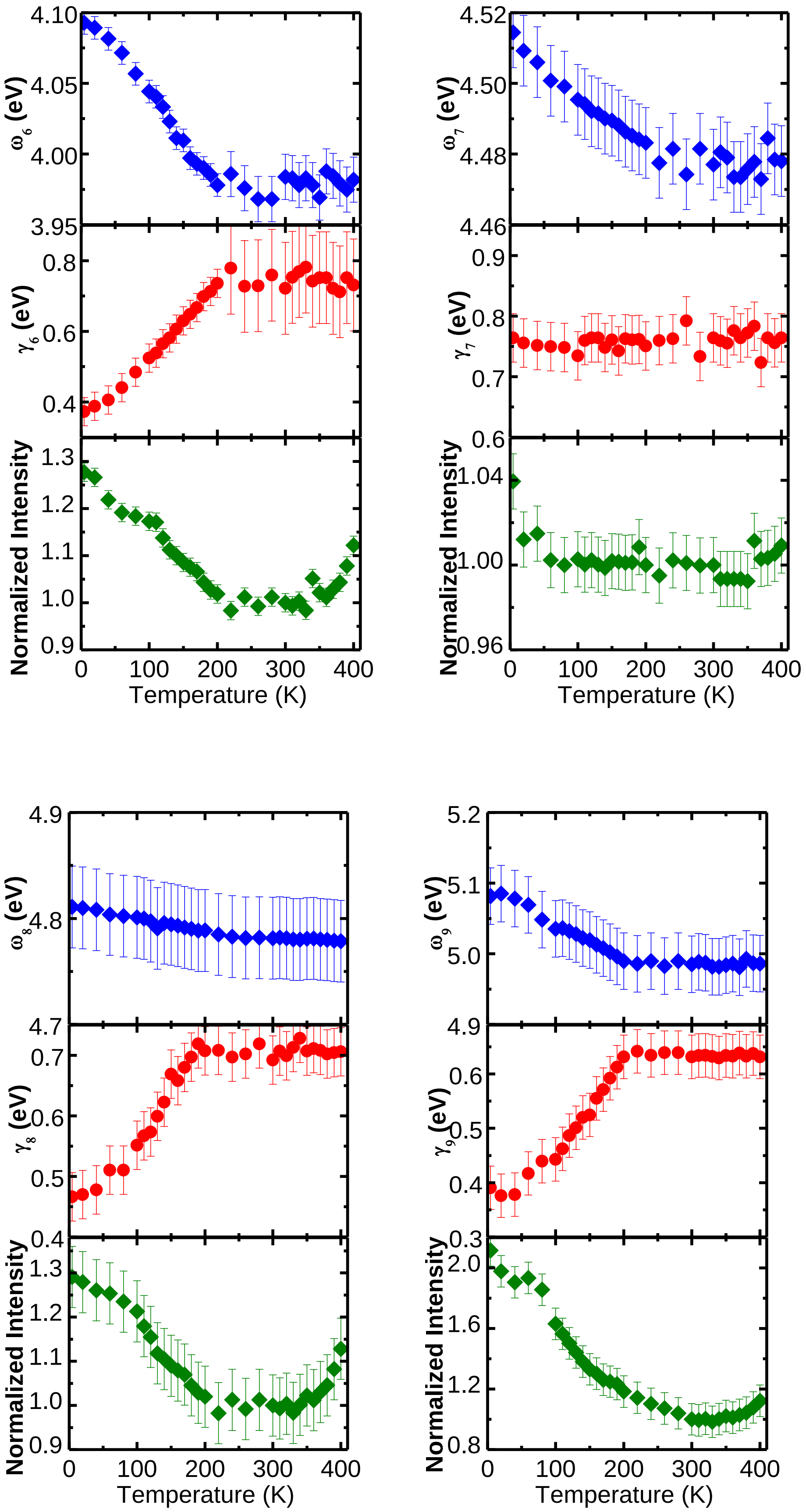
ω6 (eV)
γ6 (eV)
Normalized Intensity
Temperature (K)
ω7 (eV)
γ7 (eV)
Normalized Intensity
Temperature (K)
ω8 (eV)
γ8 (eV)
Normalized Intensity
Temperature (K)
ω9 (eV)
γ9 (eV)
Normalized Intensity
Temperature (K)

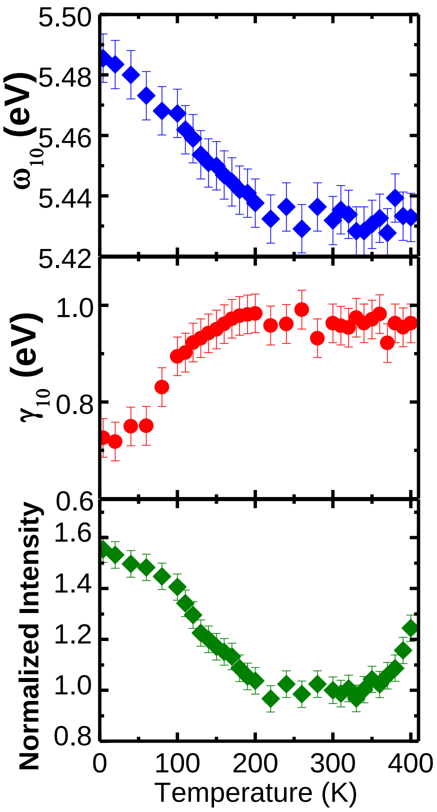

FIG. S3 Temperature dependence of peak energy, damping, and normalized intensity of high energy optical absorptions in $(MA_{0.13}FA_{0.87})PbI_3$.
